# Navigating the Central Nervous System (CNS): A Pharmacokinetic Approach to the Treatment of CNS Tumors, Glioblastoma Multiforme (GBM), in Particular

**DOI:** 10.3390/ijms26199418

**Published:** 2025-09-26

**Authors:** Dorota Bartusik-Aebisher, Jakub Tylutki, Michał Tylutki, Dominika Leś, David Aebisher

**Affiliations:** 1 Department of Biochemistry and General Chemistry, Medical Faculty, Collegium Medicum, Rzeszów University, 35-310 Rzeszów, Poland; dbartusikaebisher@ur.edu.pl; 2 English Division Science Club, Medical Faculty, Collegium Medicum, Rzeszów University, 35-310 Rzeszów, Poland; jt130173@stud.ur.edu.pl; 3 Specialist Hospital in Tarnów, 33-100 Tarnów, Poland; mictylu@gmail.com; 4 Department of Photomedicine and Physical Chemistry, Medical Faculty, Collegium Medicum, Rzeszów University, 35-310 Rzeszów, Poland; dles@ur.edu.pl

**Keywords:** glioma, glioblastoma, central nervous system tumors, blood-brain barrier, blood-tumor barrier, drug delivery, bradykinin, P-glycoprotein, convection-enhanced delivery, focused ultrasound

## Abstract

Tumors of the central nervous system (CNS) represent a unique therapeutic challenge due to the complexity of the CNS and the protective role of the blood–brain barrier (BBB). All gliomas are of glial origin, account for the majority of CNS tumors, and are classified by the World Health Organization into four grades based on malignancy. High-grade gliomas, such as glioblastoma multiforme (GBM), exhibit aggressive growth, resistance to treatment, and poor prognosis. Despite significant advancements in cancer therapy, effective drug delivery to CNS tumors remains limited due to pharmacokinetic barriers, especially the BBB, and tumor-related resistance mechanisms. This review highlights the biological characteristics of gliomas and emphasizes the current challenges in achieving effective CNS tumor treatment.

## 1. Introduction

The current state of neuro-oncology is defined by rapidly advancing diagnostic techniques and a greater emphasis on molecular profiling for more personalized treatments. Despite these strides, significant challenges remain in drug development, clinical trial design, and ensuring equitable access to care, particularly for rare and aggressive tumors like glioblastoma. New treatment modalities like interventional therapies and the strategic use of immune checkpoint inhibitors are emerging, while telemedicine expands access to care. The field faces issues with drug development due to the rarity of the disease and challenges with clinical trial design, such as a lack of enrollment and operational difficulties, notes the National Institutes of Health (NIH). A greater awareness of the need for community-based care and multidisciplinary collaboration is recognized, as formalized by the establishment of the Society for Neuro-Oncology (SNO) Community Neuro-Oncology Committee [1,2].

The central nervous system (CNS) play vital roles in our everyday lives. The main function of the spinal cord is to transfer the afferent signal from sensory organs to the brain and also to send motor commands from the brain to the peripheral parts of the body, which physiologically results in movement. Additionally, it is the brain’s duty to thoroughly process received information and give the proper response [3]. The tumor may grow in the brain itself and cause some serious problems depending on the area of the brain it grows on, but also as the brain is located in the skull, its functions may be easily disturbed as a consequence of mass growth and occupying more space, hence increasing intracranial pressure [4].

CNS neoplasms make up approximately 1% of all cancers in the human body [5]. They involve the abnormal growth of various types of cells of the brain or spinal cord, with around 75% arising from glial cells in the brain, also called gliomas. One of the World Health Organization (WHO) classifications of gliomas is based on their degrees of malignancy, where tumors can be graded between I and IV. Grade I gliomas are biologically benign tumors usually connected with neurofibromin I (NF I) mutations [6,7]. Grade II and III gliomas are a greater threat than grade I tumors. Both usually are a result of mutations in the *TP53* and *ATRX* genes. They occur more often in young adults than children. At the same time, children do not show signs of isocitrate dehydrogenase (IDH) mutation nor 1p/19q co-deletion, which are typical for these gliomas [8,9,10]. Grade IV gliomas have the worst prognoses, being more common in individuals over 65 years old.

The treatment of CNS tumors remains arduous, as we still struggle to overcome the characteristics of CNS pharmacokinetics and the tumor’s mechanisms of resistance. When fighting cancer, it is crucial to make drugs in their unbound state to achieve and maintain the optimal concentration at its site of action. In spite of the great advancements that have been established in the CNS cancer treatment field over the past few decades, we still manage to fulfill such requirements only under in vitro conditions yet fail to convey this to patients. The blood–brain barrier (BBB) has been repeatedly identified as a major pharmacokinetic obstacle to success, drastically limiting the efficacy of the drugs used in tumor therapy by blocking their passage to the tumor site [11,12,13]. Other complications are mainly caused by certain tumors’ features, like the heterogeneity or chemo-radiation resistance of cancer stem cells (CSCs) like in the example of glioblastoma multiforme (GBM) [12,13].

## 2. Glioblastoma Multiforme (GBM)

GBM, being a WHO grade IV glioma, is simultaneously the most common and most lethal primary tumor of the CNS. The incident rate of GBM is 3.21 per 100,000, and it constitutes 56.6% of all gliomas and as many as 47.7% of malignant CNS tumors [14]. Despite the maximum care given, consisting of radiotherapy, temozolomide (TMZ) administration, and immediate surgical intervention, this type of cancer still displays a dire prognosis for patients [15,16] with the overall survival (OS) for newly diagnosed GBM patients reaching 15–17 months, and only about 25% of patients living up to 2 years after being diagnosed [17,18]. Even when initially suppressed, in most cases, GBM recurs within a few months, leaving limited treatment options, as no standardized treatment for recurrent GBM has been yet introduced [11].

The exact cause of GBM remains largely unknown; however, there are some aspects recognized as risk factors for developing it. Exposure to high levels of ionizing radiation and low doses of radiation are both indicated to increase the risk for type IV glioma, with 116 GBM cases linked to radiation since the 1960s [12]. There is considerably less information about the factors that contribute to GBM development in adults. Age itself may be considered a risk factor as the peak incidence of GBM is between 55 and 84 years old [19]. Research involving Japanese survivors of atomic bomb radiation in Hiroshima and Nagasaki has shown higher rates of various gliomas [20]. There is also evidence suggesting that gliomas may run in families, although the specific genes responsible have yet to be identified. Only 5–10% of cases show a genetic tendency toward developing the disease [21]. Environmental influences such as smoking, diet, head trauma, cell phone radiation, workplace hazards, and pesticide exposure have not been definitively identified as factors increasing the chances of developing GBM.

GBM can be classified into two subgroups depending on its clinical characteristics. Primary GBM tumors arise de novo, and they develop rapidly with no signs of prior astrocytomas. The distinguishing features of primary GBM tumors include mutations and amplifications in genes coding for epidermal growth factor receptor (EGFR), high mouse double minute 2 (MDM2) expression, TERT promoter mutation, the deletion of p16, and the loss of heterozygosity (LOH) of 10q chromosome holding phosphate and tensin homolog (PTEN) [22]. On the other hand, secondary GBM tumors tend to progress from lower-grade astrocytomas with platelet-derived growth factor A and receptor alpha (PDGFA and PDGFRα) overexpression, retinoblastoma, LOH of 19q, and mutations in the *IDH1*, *TP53*, and *ATRX* genes all being the hallmark changes in these tumors [12,23].

### 2.1. Drug Efficacy

In order for a drug to be therapeutically efficient, it has to meet a few requirements. First of all, it has to be delivered to its site of action, which, in this case, is represented by tumor cells. It must be a drug that tumor cells are sensitive for and be at its active site in its active, unbound form. Moreover, there needs to be adequate exposure, which means that the drug has to be administered in high enough concentrations and for a long enough period so that it can actually result in some therapeutic effect. At the same time, the patient’s organism has to be able to tolerate the above-mentioned doses.

Generating and maintaining the appropriate exposure of an agent at the CNS tumor site require the drug to cross into the CNS. It is the first requirement to be met, as the systemically administered drug has to pass from the blood to the CNS tissue, which includes penetrating the barrier that by function prevents harmful substances from crossing into the CNS [24]. Nevertheless, despite the BBB being the most popular and most deeply studied obstacle for drugs, there are still other barriers, like the blood–tumor barrier (BTB), that share similarities with the BBB in interrupting the drug to build up a sufficient concentration at the active site. It should not be forgotten that heterogeneity, any abnormalities in the intratumoral vasculature, high interstitial pressure, and peritumoral edema are all factors that may additionally obstruct the delivery of drugs into the CNS tumor, hence making it even harder to achieve therapeutic success. Limitations in GBM treatment stem from the blood–brain barrier’s restriction of drug delivery, the tumor’s rapid growth and invasive nature, the genetic heterogeneity of cells leading to treatment resistance, the challenge of achieving gross total tumor resection, and the complex, immunosuppressive microenvironment within the tumor, all contributing to poor treatment outcomes and high recurrence rates. GBM cells are highly adaptable, characterized by rapid regeneration and the ability to migrate to distant sites, making complete surgical removal difficult and enabling recurrence. Tumors are composed of diverse cell populations with varying genetic and epigenetic traits, which contributes to treatment resistance and allows some cells to survive and regrow after therapy. Even with advanced delivery methods, obtaining enough chemotherapy for the brain tumor remains a major hurdle, with some clinical trials failing due to insufficient drug concentration [24,25].

### 2.2. Blood–Brain Barrier (BBB)

The BBB is a neurovascular structure occurring in the form of a highly selective semi-permeable membrane. In general, the BBB constitutes a few key components: endothelial cells, astrocytes, and pericytes. Wedged endothelial cells line the interior of capillary vessels and create tight junctions, simultaneously coalescing with receptors’ transporters and efflux pumps embedded in the cellular membrane to control the movement of substances between the bloodstream and the CNS [26,27,28]. The role of astrocytes in the BBB still remains debatable [29,30], with some research suggesting that they can modulate the behaviors of cerebral cells and other endothelial cells. Notwithstanding the doubtful function, it is certain that astrocytic feet, along with pericytes, surround the endothelial cells, providing support for the entire structure [31]. Pericytes are tightly connected to the basement membrane of the endothelial cells, covering nearly 100% of the surface of the cells in the CNS [32]. Such coverage and impedance to the endothelium make pericytes vital for the BBB [32,33,34]. Pericytes and endothelial cells use different signaling pathways to transfer information, thus inflecting their activity. Endothelial cells are known to release platelet-derived growth factor B (PDGFB) to bind platelet-derived growth factor receptor β (PDGFRβ) located on pericytes, resulting in a higher recruitment of stabilizing pericytes on blood vessels [35,36]. This exact communication pathway seems to be of crucial value for the barrier due to the positive correlation between the number of recruited pericytes and the number of tight junctions between endothelial cells [37].

**Figure 1:**
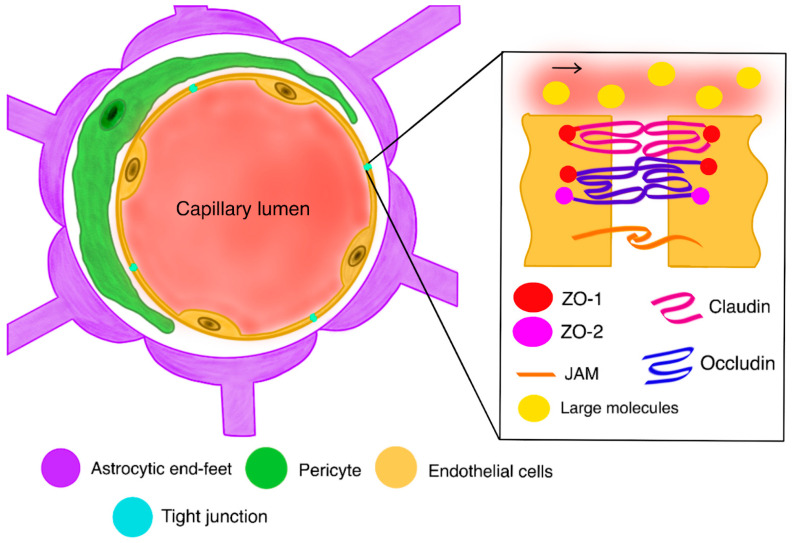
A healthy blood–brain barrier with a closer look at the mechanism of action of tight junctions; Zona-occludens 1;2 proteins (ZO-1;2); Junctional adherens molecule (JAM).

At the molecular level of the BBB, there are other indispensable elements that include tight junctions and adherens junctions (Figure 1). Tight junctions, also called occluding junctions, are an essential functional part of the barrier. Being located between endothelial cells, they close the space between cells, acting as barriers to reduce paracellular permeability and the lateral movement of membrane proteins and lipids [38,39]. Consequently, they are responsible not only for maintaining the BBB’s permeability but also for regulating the entire CNS’s homeostasis, including membrane polarization [38,39,40,41]. On the other hand, adherens junctions are critical for maintaining the integrity of the barrier and the proper assortment of protein complexes in tight junctions [42].

Physiologically, tight junctions and the proteins of adherens junctions prevent diffusion between cells, making it a necessity for the drug to cross the luminal and abluminal membranes of the endothelium for the purpose of getting into the brain parenchyma. Taking into consideration the anatomy of the BBB, several drug-related transport-limiting factors can be stated. The amount of agent penetrating the barrier is highly dependent on the agent’s molecular mass, lipid solubility, and hydrogen bonding, where small size and mass, high lipophilicity, and low hydrogen-bonding potential are advantageous in terms of passing the BBB [43]. Other factors include the metabolism, degradation, and clearance of the drug, as high systemic clearance reduces the bioavailability of the drug [44].

Since the BBB interacts with its own microenvironment by the endothelial, astrocytic, pericytic pathways, this makes the barrier dynamic [44,45]. It also means that the transport of substances to and out of the brain may be modulated in accordance with the brain’s needs. For instance, water-soluble molecules, like glucose, can quickly penetrate the BBB to satisfy the brain’s demands [44]. What is more interesting is that the same GLUT-1 and GLUT-3 transporters that facilitate glucose diffusion can also be treated as a route for the drug to enter the CNS [46]. Therefore, for drugs that rely on specific transporters, their ability to enter or exit the CNS largely depends on how strongly they bind to the carrier. Transporters that facilitate entry into the CNS, influx transporters, include organic anion transporting polypeptides (OATPs), nucleoside transporters, monocarboxylate transporters (MCTs), and putative transport systems. Efflux transporters include breast cancer resistance protein (BCRP), multidrug resistance proteins (MRPs), and P-glycoprotein (P-gp) [47]. To a great degree, resistance proteins are responsible for preventing drugs from entering the brain, but they also limit their accumulation at the site, contributing to less effective therapy [48,49].

**Figure 2:**
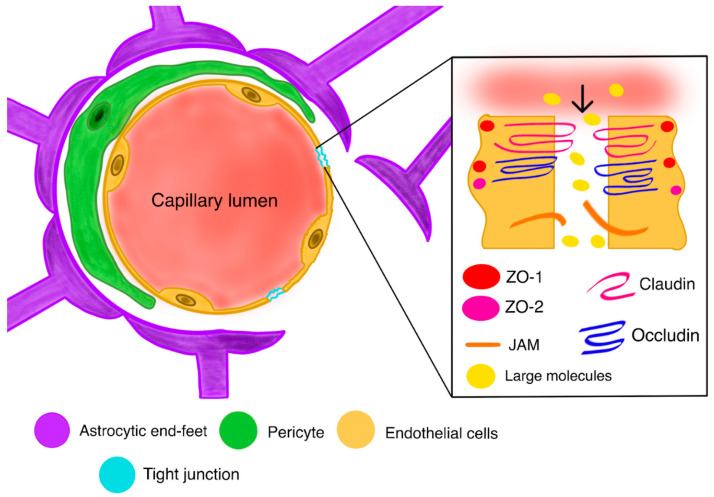
A disrupted blood–brain barrier with a closer look at the pathomechanism of the loosening of tight junctions; Zona-occludens 1;2 proteins (ZO-1;2); Junctional adherens molecule (JAM).

In CNS tumors, the BBB often becomes a victim of cancer cells (Figure 2). The inseparable elements of GBM development include the secretion of glioma-derived factors, which may actively degrade the BBB components responsible for the barrier’s integrity [50]. Reactive oxygen species (ROS), transforming growth factor beta2 (TGF-β2), and caveolin1 are primary examples of glioma-derived factors that all induce matrix metalloproteinases (MMPs). MMPs thereupon lead to the deterioration of tight junctions, which cause the migration of endothelial cells and the disruption of the vessel basement membrane and surrounding extracellular matrix (ECM) [12,51]. This and vascular endothelial growth factor (VEGF) overexpression are the main promoters of angiogenesis at the tumor site [52].

In diagnostics, disruptions of the BBB can be identified on MRI scans. Typically, a gadolinium-based contrast agent is used, which is a hydrophilic substance that leaks from blood vessels to the extracellular space (ECS) in regions of BBB impairments providing characteristic enhancement. Unfortunately, in malignant gliomas, like GBM, due to the non-homogenous character of BBB disruption, it is rare for the leakage to represent the full extent of tumor involvement [44]. There are several cases where tumor cells have been found in a normal-looking brain [53,54]. Usually, the BBB is disrupted only at the primary site of the tumor and not at the metastatic one, which is the dominant reason for GBM recurrences 2–3 cm from the site of the surgical cavity [55,56].

### 2.3. Blood–Tumor Barrier (BTB)

The BTB is a formation evolving from a disrupted BBB. It forms due to the interactions of newly formed vessels with the existing BBB [57]. As the result of glioma-derived factors and hypoxic conditions, the elements explaining the impermeability of the BBB, like tight and adherens junctions, efflux pumps, and a non-fenestrated endothelium, are all compromised in CNS tumors, including GBM [58]. Despite the deterioration of the main protective components of the BBB, the drugs that normally do not penetrate the BBB still cannot exert a therapeutic effect on the GBM tissue. Some research indicates that the heterogeneity of the BTB in GBM makes it enclose some healthy BBB regions that prevent antitumor drug accumulation [59].

The BTB, similarly to the BBB, is also targeted in MRI scans. In the regions of the BBB/BTB that show enhancements on MRI scans, the tumor blood vessels are frequently found to be abnormal, with the exhibition of dilation and poor perfusion, leading to the display of vasogenic edema [53]. From a treatment standpoint, disruptions in the BTB are key determinants of drug dispersion at the tumor site, yet as long as the degree of BTB disruption varies not only within different parts of a single tumor but also across different patients, MRI contrast enhancement alone should not be relied upon to assess whether therapeutic drugs are effectively reaching the tumor [44,53].

### 2.4. A Further Drug Route in the CNS

Attempts to circumvent or disrupt the BBB have not translated to improved survival for patients with CNS tumors [44,57]. This may be because delivering drugs faces an extra hurdle: traversing brain tissue to reach the tumor. In order for a drug to be effective, it has to not only penetrate the BBB but also remain tumoricidal at the active site for a long enough period, which is especially hard as it is known that drug concentrations decay rapidly between the site of infusion and through the brain tissue [44].

When a drug successfully crosses the blood–brain barrier, its further distribution within the central nervous system is governed by an extensive list of anatomical, physiological, and molecular factors. Once the drug enters the brain interstitial space, it meets the highly diverse and multi-layered microenvironment of the CNS, which includes the extracellular space (ECS), neuronal and glial (astrocytic, microglial, oligodendroglial) cell population, and extracellular matrix proteins. Lipophilic agents that traverse cellular structures now need to move from the lipid-bound BBB to the aqueous interstitial fluid [60].

The ECS is a part of the fluid-filled communication system between cells in the CNS. It is involved in nerve communication and the transport of drugs. This space comprises compartments filled with fluid, blood vessels, and cerebrospinal fluid (CSF)-containing spaces such as the ventricles or subarachnoid space [61]. The ECS occupies around 20% of the brain’s volume and is pocked with microscopic gaps, around 40 nm each, between cells [59]. The interstitial fluid is composed of molecules, which include negatively charged proteoglycans and hyaluronan, both being able to impair drug transport [62,63]. These matrix components not only increase the viscosity of the ECS through hyaluronan hydration [63] but also affect the diffusion of ions. The estimated diffusion coefficient for small molecules in the ECS is only about 40% of that in free solution due to these obstacles [62].

Several elements influence how medications travel in the CNS. Guided by concentration gradients, the smallest drugs passively diffuse through the interstitial fluid. The drug’s size, physical characteristics, temperature, and pressure all influence the rate of diffusion. Usually, drugs only travel a few millimeters from the infusion site [62], yet some tumor-related characteristics, like the cellular matrix, edema, ischemia, osmolarity, and cellularity, may further affect diffusion in the ECS [63].

### 2.5. Bypassing the Barriers

In the recent past, one may have observed the constantly growing interest in methods that would allow for bypassing CNS barriers or make them temporarily deactivated so that the drug could achieve some real effects. This has led to the greater use and study of various therapeutic agents, such as cytotoxic drugs, molecular therapies, or even immune-based treatments. When it comes to BBB regulation, radiation therapy, osmotic disruption, focused ultrasound, and bradykinin with its agonists were tested. Although each mentioned strategy has an impact on BBB permeability, none of them can exert specific or long-lasting effects [44,64,65,66]. Nevertheless, there are methods for, at least partially, bypassing the BBB or enhancing the therapeutic effect of drugs. Among others, methods applicable in the treatment of GBM include high-dose systemic therapy, the disruption of the BBB, the inhibition of efflux transporters, and regional therapy.

### 2.6. High-Dose Systemic Therapy

In the past, GBM treatment was based on the surgery of the tumor site supported by radiotherapy. After a large, randomized trial (NCT00006353), it has become clear that temozolomide (TMZ) administration is beneficial for the OS of patients [66]. The current standard treatment for GBM tumors consists of surgery followed by concurrent radiation therapy and daily TMZ, later followed by another six cycles of TMZ. Since the time when chemotherapy enriched the way cancers are dealt with, it has been the primary goal for clinicians to achieve the highest dose of the active agent in the shortest possible time. These aspirations resulted from the assumptions that an increase in a drug’s concentration in the blood would proportionally increase its entry to the CNS. However, this idea is reasonable only for the drugs that cross the BBB by passive diffusion or for those whose carrier mechanisms are not saturated. The administration of high doses of such drugs would cause them to distribute more evenly in the CNS, including deep brain tissues and perivascular areas, regardless of CSF flow direction or rate [44]. TMZ acts on glioblastoma multiforme (GBM) by undergoing hydrolysis to form the active metabolite methyltriazen-1-yl imidazole-4-carboxamide (MTIC), which releases a reactive methyl group that binds to DNA at guanine and adenine bases. This DNA methylation causes mismatches that lead to cell cycle arrest and apoptosis (programmed cell death) during DNA replication. Prodrug to Active Metabolite: The oral prodrug temozolomide (TMZ) is absorbed and then undergoes hydrolysis at physiological pH to form the active metabolite MTIC. DNA Alkylation: MTIC then degrades to produce a reactive methylating species. Methyl Group Transfer: This species transfers a methyl group to guanine (O6 and N7 positions) and adenine (N3 position) bases in the DNA. DNA Damage: The resulting O6-methylguanine (O6-MG) is the primary cytotoxic lesion. Cell Cycle Arrest and Apoptosis: During DNA replication, the DNA mismatch repair (MMR) system attempts to correct these O6-MG adducts. When the damage is too extensive, this process can induce single- and double-strand breaks, leading to cell cycle arrest and triggering apoptotic (programmed cell death) pathways [67,68,69,70].

Only a limited number of clinical trials, involving a small number of patients, have explored the effects of an altered TMZ regimen, for example, NCT00304031. Generally, trials aim to determine whether the modified dosing of TMZ could raise the drug’s cumulative dose and effectiveness by reducing O6-methylguanine-DNA methyltransferase (MGMT) levels, all without an increase in toxicity. Many changes have been implemented into the standard regimen. Alternative dosing includes one week on/one week off and nearly constant TMZ dosing in 21/28-day cycles [71,72,73]. All the trials indicate that a dose-dense TMZ regimen is effective at reducing MGMT levels, but at the same time, the observed outcomes suggest that MGMT depletion appears to offer only limited benefit in the treatment of malignant gliomas.

### 2.7. BBB Disruption

The disruption of the BBB is a well-established method aimed at increasing the bioavailability of the drug for the CNS. This method can be performed in many ways, including hyperosmotic solution infusion, the administration of vasoactive substances, or external stimulation, like ultrasound.

The infusion of hyperosmotic solution, such as mannitol, is the most clinically recognized method of all the ones mentioned. Hyperosmotic solution infusion results in the shrinkage of endothelial cells, hence the opening of tight junctions [74], which results not only in the desired increased permeability of the BBB but also higher CNS toxicity [75]. There were, and still are, many clinical trials trying to evaluate the therapeutic effect of the intra-arterial infusion of mannitol combined with chemotherapy in GBM management (NCT02800486, NCT00968240, NCT01180816, NCT01269853, NCT02285959, NCT05271240, NCT01811498). To date, conclusions concerning the safety of the technique are debatable, with some research suggesting that therapy enhanced by BBB disruption with the use of mannitol is safe and well tolerated by patients and others being terminated due to toxicity and neurological complications [76]. In some studies, the initial results have shown a reduction in both the median area and volume of the tumor [75,76,77,78]. Despite the fact that these are only phase I//II trials, the results are promising, giving hope for better GBM treatment in the future. The intravenous (IV) administration of bevacizumab is the standard route for bevacizumab. However, relevant comparisons are made between the intra-arterial (IA) and intraperitoneal (IP) administration of bevacizumab.

Bevacizumab (IV) is typically administered as a monoclonal antibody that blocks a growth factor (VEGF) to inhibit tumor growth and blood vessel formation.

In some cases, bevacizumab is administered via other routes like the intra-arterial (IA) or intraperitoneal (IP) route. For example, one study showed significant cost savings with intra-arterial bevacizumab compared to IV administration for recurrent glioblastoma, despite the higher dose used. Intravitreal Use: For conditions affecting the eye, such as diabetic macular edema (DME), a different bevacizumab product might be given directly into the eye (intravitreal), rather than systemically. However, for DME, comparisons show that intravitreal ranibizumab often leads to greater improvements in visual acuity than intravitreal bevacizumab [79,80].

**Figure 3:**
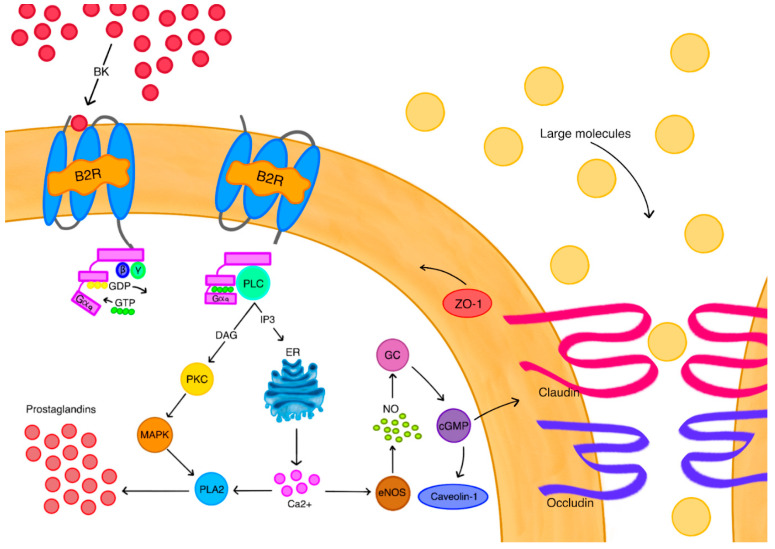
Role of bradykinin as biochemical modulator of tumor BBB permeability; entire signaling pathway is described below.

In GBM tumors, the tumoral BBB disruption is heterogeneous, meaning that some tumor cells located behind the intact BBB regions remain indifferent to therapy. Therefore, relying solely on drug delivery to areas with a pathologically disrupted BBB is likely to lead to missed tumor cells, resulting in quick recurrence. To avoid those consequences, it is crucial to develop an approach allowing for the effective targeting of the entire tumor area. The administration of vasoactive substances can indeed affect the way that CNS tumors are managed. Nowadays, there are several methods of biochemical BBB modulation tested, including the use of an ATP-sensitive potassium channel (K_ATP_ channel), Calcium-activated potassium channel (K_Ca_ channel), Phosphodiesterase 5 (PDE5), Bradykinin type 2 receptor (B2R), Adenosine 2A receptor (A2AR), Papaverine, and microRNAs (Figure 3).

For now, the K_ATP_ and K_Ca_ channels are being studied in terms of minoxidil sulfate and NS1619 transport, respectively, with the K_ATP_ channel showing a decreasing effect on occludin and claudin 5 concentrations in tight junctions. Although both methods exhibit promising results, with an enhancement in vesicular transport in the first place [80,81,82,83,84,85,86,87], all trials are still in the preclinical phase. A much more prevalent method in the clinic involves acting on B2R with the use of bradykinin and its agonists [88]. Labradimil is a peptide designed for selectively binding to B2R and has a longer plasma half-life than bradykinin, without the loss of bradykinin-like effects [89].

Bradykinin demonstrates a permeability-increasing impact on the BBB [89,90,91,92,93,94,95,96,97,98,99]. After bradykinin, or its agonist, binds to B2R on endothelial cells, the replacement of GDP by GTP occurs on the Gαq subunit, all complemented by the dissociation of the Gαq and Gβγ subunits. Gαq activates phospholipase C (PLC), which results in the release of the second messengers: inositol trisphosphate (IP3) and diacylglycerol (DAG). IP3 induces the release of calcium ions from the endoplasmic reticulum (ER). DAG activates protein kinase C (PKC), which then stimulates a mitogen-activated protein kinase (MAPK). Both MAPK and calcium ions induce the secretion of phospholipase A2 (PLA2), the higher activity of which allows for the synthesis of prostaglandins, which later on results in magnified vasodilation [97,98]. Calcium ions also induce the secretion of endothelial nitric oxide synthase (eNOS) which produces nitric oxide (NO). NO directly stimulates guanylate cyclase (GC), leading to the production of cyclic guanosine monophosphate (cGMP). The release of cGMP results in higher caveolin-1 synthesis, which promotes vesicular transport [99], but also decreases the expression of zonula occluden-1 (ZO-1) proteins, occludin, and claudin 5 [100,101], which are all essential for the integrity of tight junctions. There is evidence, derived from preclinical trials on glioma patients, that labradimil is able to permeabilize the BTB to the extent that higher doses of chemotherapeutics can be delivered to tumors without the toxicity typical for dose escalation [90]. Inevitably, the impact of B2R expression has also been studied, with some authors suggesting that B2R expression levels in human glioma could be potentially used for monitoring treatment progress in patients whose treatment involves bradykinin modulation [98]. For GBM tumors, there are also studies that examine the impact of B1R on tumor cells. Research on rodent models shows that B1R promotes the progression of GBM by enhancing the expression of adhesion molecules, like ICAM-1 and VCAM-1, but also increasing the migration of GBM cells [98]. The receptor also stimulates the production of tumor-promoting cytokines, like IL-8 [99], and upregulates heme-oxygenase-1, making GBM cells less vulnerable to oxidative stress. Besides this, it was found to interfere with death-ligand 1 expression in tumor cells and macrophages, making them resistant to T-lymphocyte immunity [101]. Summing up all these findings, bradykinin and its agonists are useful tools in circumventing barriers by permeabilizing the BBB and BTB. Moreover, they are still poorly understood, yet there is a growing interest in the role of bradykinin receptors in pathogenesis and monitoring GBM progression. 

Another method focuses on utilizing focused ultrasound (FUS) to affect BBB permeability. Ultrasonic disruption is a non-invasive technique that can exert various impacts depending on the selected parameters of the device. In attempts to disrupt the BBB, FUS is typically used with microbubbles, which are ultrasound imaging contrast agents. Microbubbles are tiny, 1–10 μm, shells containing gas that concentrate the ultrasound field to the walls of the blood vessels [102]. As the result of these interactions, tight junctions are widened, and transcellular transport mechanisms are activated with little if any effect on the parenchyma [103]. When it comes to CNS cancers, in the last decade, there were hundreds of clinical studies conducted on the practical use of FUS in malignant CNS tumor therapy, including mainly recurrent GBM. Research shows that there were no significant adverse effects reported during the procedure, with additional benefits for drug delivery [104].

## 3. Discussion

### 3.1. Inhibition of Efflux Transporters

Efflux transporters are embedded in the luminal and basal membrane of the endothelial cells of the BBB, constantly facilitating the removal of even the smallest metabolites from the CNS, including therapeutic agents [105]. The most studied are the transporters encoded by ATP-binding cassette families B1 and G2 (ABCB1; ABCG2), like P-pg, that are responsible for the efflux of non-polar and less amphiphilic molecules, which are involved in therapeutics such as TMZ and topotecan [106]. Consequently, it comes as no surprise that the absence of these transporters results in better penetration and the better maintaining of drug concentration in the CNS of mice [107].

In humans, it is possible to not get rid of efflux pumps but to inhibit them, enhancing the accumulation of chemotherapeutics at the tumor site. Inhibitors include cyclosporin A, valspodar, elacridar, and tariquidar, which act on efflux pumps in different ways, preventing their activity or blocking their binding site [108]. Cyclosporin A and valspodar failed in clinical trials due to their weak ABCB1 binding affinity [109]; however, multiple rodent studies [109] have shown the higher specificity and low toxic profile of elacridar. Despite the promising results, further research discovered that elacridar exhibits serious toxicity when combined with chemotherapeutics [110]. Unfortunately, the current knowledge on efflux transporter inhibitors does not allow us to overcome CNS barriers, as it only allows us to conquer multidrug resistance by the modulation of pumps in solid tumors [111]. Nevertheless, even if efflux regulation is successful, the toxicity caused by these inhibitors limits their use in the clinic.

### 3.2. Regional Therapy

Regional therapy is based on bypassing the BBB, increasing the drug’s therapeutic effect, and reducing its toxicity by delivering it directly to the action site. Intratumoral treatments occurred as an intuitive idea that the local administration of a chemotherapeutic may achieve greater success than standard, more pharmacokinetic-dependent, systemic therapy.

There are plenty of methods that have been already tested in clinical trials for high-grade gliomas; these include injections into the resection cavity, administration through an intraoperatively placed reservoir catheter, and also low-flow infusion for convection-enhanced delivery (CED) [112]. Despite the promising results obtained in clinical trials and the fact that these methods can actually bypass the BBB, their effectiveness is still limited by several factors. In regional administration, the major problem is achieving an adequate distribution of drugs in the parenchyma, mainly due to the fact that this approach is based on simple diffusion. As a result, only the nearest surrounding tissue is effectively exposed to the agent [113,114].

To a great extent, diffusion in a tissue is determined by concentration gradients. During the simple administration of a drug to the active site, it diffuses to the neighboring tissue, minimizing the differences in concentrations and causing the cessation of further diffusion. However, this can be overcome by pressure gradient-reliant methods like CED. The entire idea of CED is to apply pump-connected catheters to the patient’s skull and with their use, maintain the pressure gradient during drug delivery [115]. The controlled pressure gradient allows for the improved distribution of a great volume of the therapeutic agent at the tumor site, limiting the exposure of healthy tissue [116,117]. There are 17 trials with published studies concerning GBM and recurrent GBM treatment with the use of CED [79]. Among them, there is only one completed phase III clinical trial (NCT00076986), which involved using an experimental drug, IL13-PE38QQR, on patients with recurrent GBM. The drug is composed of human interleukin 13 (IL-13) and pseudomonas exotoxin. The mechanism of action is simple; when IL-13 binds its IL-13 receptor α on tumor cells, it induces the entrance of a bacterial toxin to the cells, which then promotes cell death [118].

Although all of the 17 clinical trials demonstrated safety and efficiency compared to systemic therapy [79,119], this does not mean that CED remains without its own challenges and limitations. Despite modern algorithms that improve the placement of catheters and their optimal designs, there are still incidents of drug refluxes, which decrease the effectiveness of CED significantly [120]. In the case of GBM, there is also a problem with the tumor shape. As the vast majority of gliomas are non-spherical, covering the tumor with a therapeutic agent properly and effectively via CED becomes a hurdle [121]. Also, the local control provided by CED is not necessarily an optimal and long-term solution for patients with deep, diffuse, white-matter lesions, due to their tendency toward distant recurrences [122].

A pharmacokinetic approach to CNS tumors, including glioblastoma multiforme treatment, involves optimizing drug delivery and efficacy by understanding how drugs are absorbed, distributed, metabolized, and excreted in the brain while overcoming challenges like the blood–brain barrier (BBB) (Table 1). The standard of care for GBM is a combination of surgery, radiation therapy, and temozolomide (TMZ) chemotherapy. Pharmacokinetic principles are crucial for improving BBB penetration and the targeting of drugs to the tumor [117].

Challenges in GBM therapy, particularly for immune and vaccine-based treatments, include the immunosuppressive tumor microenvironment (TME) caused by metabolic reprogramming and a lack of T-cells, the BBB that impedes drug delivery, tumor heterogeneity, the presence of cancer stem cells which evade treatment, and the host immune response that can promote tolerance rather than antitumor activity. Overcoming these obstacles may involve combining therapies, using personalized vaccines, or developing new approaches to penetrating the BBB and reprogramming the TME. Metabolic reprogramming: GBM cells utilize aerobic glycolysis (the Warburg effect), creating an acidic TME that hinders the function of immune cells like cytotoxic T-lymphocytes. Immune cell suppression: Tumors are infiltrated by immunosuppressive cells such as tumor-associated macrophages (TAMs) and T-regulatory cells (Tregs), which promote tumor growth and immune evasion. The low immunogenicity of GBM tumors makes them considered “cold tumors” due to their inherent lack of strong immune cell infiltration and the limited activation of the host immune response [132,133].

Oncolytic herpes simplex viruses (oHSVs) are a promising glioblastoma (GBM) treatment, with the G47Δ virus recently approved in Japan for recurrent/residual GBM, demonstrating improved survival rates and a good safety profile. oHSVs are genetically engineered to specifically infect and replicate in cancer cells, triggering antitumor immune responses and enhancing the effectiveness of other therapies like chemotherapy and radiation. Challenges include delivering the virus effectively without multiple surgical interventions, though high-frequency ultrasound may offer a less invasive solution [134]. Also, Oncolytic Zika Virus (ZIKV) works by inducing a pro-inflammatory response and promoting the infiltration and activation of T-cells within the tumor, which leads to tumor inhibition and improved survival [135]. ZIKV preferentially infects and kills GBM stem cells (GSCs), which are resistant to conventional therapies. Clinical trials are necessary to fully evaluate the safety and efficacy of ZIKV-based therapies in human patients [136].

## 4. Conclusions and Future Direction

Despite the effort and how deeply we delve into the intricacies of tumor biology and CNS pharmacokinetics, overcoming GBM still remains a formidable clinical challenge. The unique architecture and physiology of the CNS, dominated by the protective yet very restrictive BBB and the evolving BTB, substantially hinder the delivery and efficacy of many systemic therapeutic agents. Natural CNS barriers, when combined with the presence of tumor stem cells, tumor advanced and dynamic microenvironments, and tumor heterogeneity, make conventional pharmacological techniques become futile.

A variety of strategies aim to circumvent or adjust these hindering barriers; these include the following: high-dose systemic therapy, the disruption of the BBB, the inhibition of efflux transporters, and regional delivery methods such as CED. All methods do have their set of benefits and limitations. For instance, BBB disruption increases drug availability by permeabilizing the barrier; however, as permeability increases, so does neurotoxicity. In the case of CED, this method offers targeted delivery and great control in terms of the administered concentrations, yet it is still limited by technical challenges and tumor morphology.

Despite the hope provided by earlier-stage clinical trials, especially those incorporating BBB modulation or targeted drug delivery, breakthroughs are still elusive. Nevertheless, focused studies on the molecular and architectural features of the CNS and tumor microenvironments, paired with innovations in drug delivery mechanisms and biomarker systems, stand to improve outcomes.

To conclude, addressing the pharmacokinetic challenges inflicted by CNS tumors needs sophisticated parameters for the design of drugs, careful considerations for their delivery, and detailed knowledge of the CNS blueprint, which are collectively multifactorial. If such comprehensive approaches are adopted, fighting malignant CNS tumors will become a realizable target. Limitations in GBM treatment stem from the blood–brain barrier’s restriction of drug delivery, the tumor’s rapid growth and invasive nature, the genetic heterogeneity of cells leading to treatment resistance, the challenge of achieving gross total tumor resection, and the complex, immunosuppressive microenvironment within the tumor, all contributing to poor treatment outcomes and high recurrence rates.

Promising advancements in GBM treatment include nanotherapy for targeted drug delivery, immunotherapies like CAR T-cell therapy and vaccines to boost the immune response, and oncolytic virotherapy using viruses to destroy tumor cells. Other approaches involve using focused ultrasound to open the blood–brain barrier, biopolymer-based interstitial therapies for sustained drug release, and precision medicine guided by the advanced molecular and genetic profiling of the tumor.

Future GBM treatment perspectives include immunotherapies (like CAR T-cells and vaccines), targeted therapies, oncolytic virotherapy, and technological advances such as focused ultrasound and novel drug delivery systems for overcoming the blood–brain barrier. Personalized approaches, integrating genomic data and patient-derived organoids, are crucial for tailoring therapies and improving outcomes for this aggressive and recurrent brain tumor.

## Figures and Tables

**Table 1:** Key challenges in CNS drug delivery.

No	Key Challenges in CNS Drug Delivery	Description	References
1	Blood–Brain Barrier **(BBB)**	The BBB, a protective barrier, restricts the entry of many systemic drugs into the brain, significantly limiting therapeutic options.	[118]
2	**Tumor Infiltration**	GBM infiltrates the brain extensively, making complete surgical removal difficult and requiring systemic treatments to reach microscopic tumor cells.	[119]
3	**Tumor Heterogeneity**	GBM is highly heterogeneous, with different cell populations having varying responses to treatments, leading to drug resistance.	[120]
4	**Drug Resistance**	Mechanisms like the DNA repair enzyme O6-methylguanine methyltransferase (MGMT) can counteract the effects of chemotherapy, reducing drug efficacy.	[121]
5	**Targeted Drug Delivery**	Developing methods to increase drug concentrations at the tumor site while minimizing systemic side effects is key.	[122]
6	**Modifying Drug Properties**	Researchers are investigating drugs with better lipophilicity and lower molecular weight to enhance BBB penetration.	[123]
7	**Drug Delivery Systems**	Utilizing Nanotechnology, such as nanoparticles, can encapsulate drugs, protecting them from degradation and aiding in crossing the BBB.	[124]
8	**Disrupting the BBB**	Methods for temporarily disrupting the BBB, like using focused ultrasound, can increase drug delivery to the tumor.	[125]
9	**Targeting Tumor-Specific Transporters**	Some tumors in the brain have upregulated transporter proteins that can be exploited for targeted drug delivery.	[126]
10	Current and Emerging Treatments	**Temozolomide (TMZ)** **Tumor-Treating Fields (TTFields)** **Molecularly Targeted Therapies** **Immunotherapy** **Drug Repurposing**	[127,128,129,130,131]
